# Case of Vesico-Vaginal Fistula

**Published:** 1849-03

**Authors:** Henry H. Smith

**Affiliations:** Philada.


*Case of Vesico-vaginal Fistula. Relieved by the ordinary hare-lip operation.* By HENRY H. SMITH, M. D., Philada.

Bridget Mc——, æt. 28 years, was taken in labour about 1 A. M. of September 18th, 1847. The labour ceasing, and the head being locked, her medical attendant applied the forceps at 2 A. M. of 19th, and delivered a large still-born child.

For several hours after delivery, the catheter was used; but on the 21st, the urine began to escape freely through the vagina. She now soon became excoriated; her water wet every thing about her; ran into her shoes; wet the floor wherever she stood, the bed, &c., &c., and rendered her situation most distressing.

On December 28th, 1847, she presented herself to me for relief from the chafing, &c.

On examining the parts I found considerable inflammation of the vulva, perineum, anus and thighs, and a free and constant discharge of urine from the vagina; none passing through the urethra. The finger, on being introduced into the vagina passed directly up into the bladder, which was contracted so as scarcely to hold a walnut, whilst a catheter in the urethra could be passed backwards only about one inch, before it came out into the vagina. There was, therefore, no difficulty in recognizing the existence of a vesico-vaginal fistula, accompanied with a considerable loss of substance.

Subsequent examination with the speculum uteri showed the opening to be about the size of a Spanish quarter dollar piece; that it existed at the neck of the bladder, extending backwards towards the orifices of the ureters, and forwards to within one inch of the external opening of the urethra, and that it occupied very nearly the position of the vesical triangle.

The anterior half of the opening was rounded, thick and callous, but the posterior was much thinner and covered by the mucous membrane of the bladder, a small fold of which forming a vascular roll, projected into the vagina.

The anterior lip and neck of the uterus were adherent to the front of the vagina, close to the edge of the fistula. After applying soothing remedies, and overcoming the external irritation, it was determined to try an operation, although its success seemed very doubtful

Accordingly, after freely dilating the urethra by the use of large bougies, I proceeded, January 17th, 1848, at the suggestion of Dr. Wm. E. Horner, to operate as follows: The woman being placed with her hands and feet tied in the ordinary position for lithotomy, was brought to the edge of the bed before a strong light, and held in that position by Drs. Taliaferro, of Va., and Benton, of N. Y. A half speculum, open on the top, was next placed in the vagina, and one of Lisfranc's long double hooks inserted into the posterior part of the neck of the uterus, (as advised by Jobert,) so as to draw it fairly down to within about two inches of the os externum, thus diminishing the size of the fistulous opening by relaxing the vagina.

A piece of soft pine wood about the size of the fore finger, convex on one side and flattened on the, other, was then passed into the urethra, with its flat side next to the vagina, and carried back into the bladder, so as to depress the edge of the fistula. Upon this, I cut off the posterior lip of the opening, partly by a vertical bladed knife made for the occasion, and resembling somewhat an ordinary hoe, and partly by a long bladed scalpel. By elevating the handle of the stick in the urethra, the anterior lip of the fistula was also readily depressed and its edges pared off, the paring being freely continued into the angles of the fistula. Four long hare-lip pins, slightly bent, an fastened into an acupuncturation needle holder, were now passed one after the other, through the anterior lip of the fistula from below upwards, and then through the posterior lip from above downwards, a part of the pin being of course in the urethra. Four figure of 8 ligatures thrown around the pins, now closed the fistulous opening so perfectly that a fine probe could not be made to pass it, except at a spot between the two central pins, where the strain was evidently greatest. A catheter introduced into the bladder prevented, however, any escape of urine through the wound, and the vagina being filled with charpie, and the patient kept on her abdomen, the proper course of the urine was soon restored, it continuing to flow entirely by the catheter and not through the vagina.

Slight fever in the evening.

*January 8th.*—Very comfortable—urine escapes clear through the instrument.

*14th.*—Removed the pins cautiously, but noticed a slight escape of urine per vaginam—replaced catheter by a fresh one.

*20th.*—Examined with the speculum and found union at the angles and well on to the centre pin, where it was deficient—the opening is, however, diminished at least two-thirds—touched with nitrate of silver and continued catheter.

*27th.*—Obliged to remove catheter on account of irritation; the patient can now hold near a tumbler of urine—especially when on her abdomen. By continued use of the caustic the fistula diminished rapidly, until not larger than the end of the little finger, but at this time she suddenly left town and I lost sight of her.

*January 27th,* 1849.—I again heard of my patient through the friend who nursed her during the operation, and learned "that she is not troubled in the least with her water, and is again living with her beau."

Owing to the great loss of substance, the approximation of the sides of the fistula would have been impossible, except by the aid of the hare-lip suture.

